# Dataset of tugHall simulations of cell evolution for colorectal cancer

**DOI:** 10.1016/j.dib.2021.106719

**Published:** 2021-01-11

**Authors:** Iurii S. Nagornov, Jo Nishino, Mamoru Kato

**Affiliations:** Division of Bioinformatics, Research Institute, National Cancer Center Japan, Tokyo 104-0045, Japan

**Keywords:** Approximate Bayesian computation, Colorectal cancer, Evolution of cancer cells, Dataset of tugHall simulator, Genome and cancer hallmarks relationship

## Abstract

Dataset contains results of multiple parallel calculations using the **tugHall** simulator. Output data of simulations are variant allele frequencies for four genes (APC, KRAS, TP53, and PIK3CA) related to colorectal cancer. During each simulation **tugHall** stochastically reproduces Darwinian evolution for cancer cells and calculates clonal heterogeneity. The probabilities of stochastic processes depend on a correspondence matrix between genome information and cancer hallmarks. As a result, **tugHall** records variant allele frequencies for the final stage of evolution. The number of trials is several million to get rich statistics of stochastic processes. These data can be used for approximate Bayesian computation and other statistical methods to get personalized coefficients for patients with colorectal cancer. The procedure of usage data is explained in our paper [Bioinformatics, 36, 11 (2020) 3597] in which the part of these data was used.

## Specifications Table

**Table:** 

Subject	Bioinformatics
Specific subject area	Colorectal oncology and Mathematical modeling
Type of data	Statistical simulation for variant allele frequencies of clonal evolution of colorectal cancer
How data were acquired	The dataset is a set of results of 9.6 millions of simulations using *tugHall* simulator. To get a large number of simulations we used the resources of the supercomputer “SHIROKANE” of the Human Genome Center of the University of Tokyo [https://supcom.hgc.jp].
Data format	Simulation data (tabular format), Modeling workflow (figure)
Parameters for data collection	Identification number of simulation, names of models, initial conditions, the format of input parameters, weights for hallmarks and genes, compaction factors, probabilities of stochastic processes, as well as results of simulations (variant allele frequencies, numbers of primary tumor cells and metastatic cells, last time-step, number of clones).
Description of data collection	Data were collected as a result of multiple simulations. Parallel calculations with 40 nodes and 960 cores were used to perform 9,600,000 trials for 4 models, 3 types of initial clones, and 2 types of input data. In total there are 24 combinations with 400,000 trials for each.
Data source location	Institution: National Cancer centre Japan, Research Institute, Division of Bioinformatics City/Town/Region: Tokyo Country: Japan
Data accessibility	Nagornov, Iurii; Nishino, Jo; Kato, Mamoru (2020), “Dataset of tugHall simulations of cell evolution for colorectal cancer ”, Mendeley Data, V1, doi: 10.17632/spszxd8r3z.1 http://dx.doi.org/10.17632/spszxd8r3z.1
Related research article	Iurii S. Nagornov, Mamoru Kato, tugHall: a simulator of cancer-cell evolution based on the hallmarks of cancer and tumor-related genes, Bioinformatics, Vol. 36, N 11 (2020) 3597–3599. doi: https://doi.org/10.1093/bioinformatics/btaa182

## Value of the Data

• Dataset has the usage potential to predict the target gene for the treatment of colorectal cancer in personalized medicine.
• Dataset can be useful in the field of bioinformatics and biostatistics to choose the target gene, and it is complementary to survival analysis to improve the probability of survival for a patient using genome information.
• Approximate Bayesian computation allows us to extract the weights of the relations between driver genes and cancer hallmarks in the tugHall model for a particular patient. Using the personalized weights, it is possible to predict which gene should be blocked to stop cancer development.
• The accuracy of the prediction depends on the size of the dataset. That's why it has results of several million simulations, and it will continue growing in the future.

## 1 Data Description

The dataset provides results of 9.6 million calculations using the tugHall simulator. Output data of simulations are variant allele frequencies (VAF) for four genes APC, KRAS, TP53, and PIK3CA related to colorectal cancer [1]. During each simulation tugHall stochastically reproduces Darwinian evolution for cancer cells and calculates clonal heterogeneity [2]. Calculations of VAF were performed at last time-step of simulation as well as statistical data like numbers of the primary tumor and metastatic cells, final time-step and number of clones. The VAFs were calculated for two cases. First one is VAF for all cell in the simulation pull, second is VAF only for primary tumor cells. These results are divided into two files with the same structure (data_base_MODELS_ALL.txt and data_base_MODELS_PRIMARY.txt respectively). The file data_base_STATISTICS.txt contains the statistical data of simulations. In total the dataset has 8 files: 3 files with output data, 4 files with input data and file with analytic data (Table 1). Table 1 shows the short description of each file as well it's size.

**Table 1:** Description of files in the dataset.

File Name	Size	Content of the file
**Analyze_MODELS_PRIMARY.txt**	1 Kb	file with **analytic** data of simulations: number of successful simulations and simulations with non zero output for each type of simulation (corresponds to Table 2).
**data_base_MODELS_ALL.txt**	700.7 Mb	**output** file with VAF for **all** cells (including metastatic cells) at last time-step.
**data_base_MODELS_PRIMARY.txt**	166.1 Mb	**output** file with VAF for **primary tumor cells** only at last time-step.
**data_base_STATISTICS.txt**	113 Mb	**output** file with statistical data for each simulation like number of clones, numbers of primary tumor and metastatic cells, and time of simulation's stopping.
**Compaction_Factor_Continuous_ALL.txt**	16.5 Mb	**input** file with data of compaction factors for each simulation for **continuous** type of data.
**Compaction_Factor_Discrete_ALL.txt**	10.3 Mb	**input** file with data of compaction factors for each simulation for **discrete** type of data.
**Initial_parameters_Continuous_ALL.txt**	68.5 Mb	**input** file with input data for each simulation with **continuous** type of parameters.
**Initial_parameters_Discrete_ALL.txt**	46.9 Mb	**input** file with input data for each simulation with **discrete** type of parameters.

Table 2 shows analytical data for different types of simulations. Each simulation can finish with two possible cases: the first one is when all cells died and without any output data (unsuccessful), another case is a simulation with output data (column “success” in Table 2). The successful simulations consist of two subsets: with zero output for all VAFs and with non-zero VAF at least for one gene (column “non_Zero” in Table 2). Hereinafter, VAF means VAF for primary tumor cells. In total the dataset has 1,706,179 records with 284,674 non-zero outputs from 9,600,000 trials of 24 types of simulations.

**Table 2:** Analytical data for different types of simulations.

name_weights	name_model	name_init	success	non_Zero
Discrete	STRONG	Mutated_cell	6757	6672
Discrete	STRONG	Thousand_cells	17,308	17,290
Discrete	STRONG	Mutated_cell_in_Thousand_cells	34,602	34,059
Discrete	WEAK	Mutated_cell	279,994	9335
Discrete	WEAK	Thousand_cells	155,889	44,533
Discrete	WEAK	Mutated_cell_in_Thousand_cells	217,965	86,083
Discrete	CF_STRONG	Mutated_cell	606	598
Discrete	CF_STRONG	Thousand_cells	3301	3300
Discrete	CF_STRONG	Mutated_cell_in_Thousand_cells	6962	6832
Discrete	CF_WEAK	Mutated_cell	99,560	719
Discrete	CF_WEAK	Thousand_cells	21,741	6383
Discrete	CF_WEAK	Mutated_cell_in_Thousand_cells	48,347	19,949
Continuous	STRONG	Mutated_cell	60	60
Continuous	STRONG	Thousand_cells	142	142
Continuous	STRONG	Mutated_cell_in_Thousand_cells	10,516	10,476
Continuous	WEAK	Mutated_cell	296,449	16
Continuous	WEAK	Thousand_cells	137,629	10,822
Continuous	WEAK	Mutated_cell_in_Thousand_cells	203,408	20,982
Continuous	CF_STRONG	Mutated_cell	2	2
Continuous	CF_STRONG	Thousand_cells	12	12
Continuous	CF_STRONG	Mutated_cell_in_Thousand_cells	459	453
Continuous	CF_WEAK	Mutated_cell	104,189	3
Continuous	CF_WEAK	Thousand_cells	17,573	1417
Continuous	CF_WEAK	Mutated_cell_in_Thousand_cells	42,708	4536

Correspondence between input and output data is connected with an identification number of simulation (ID_Simulation). Table 3 shows the first several rows of the dataset for the results of simulations. It has information about the names of models, initial cells, the format of input parameters, and identification number of simulation as well as results of simulations. The results are summary statistics such as variant allele frequencies that are used in bulk-cell signaling data [1] and represented in the descending order of VAF for each driver gene. The dataset has the 5 largest values of VAF for each gene (APC, TP53, APC, and PIK3CA) but in Table 3 we have shown only the first values of VAFs for each gene. The structure of data in the file “data_base_MODELS_ALL.txt” is the same.

• name_weights - “Discrete” or “Continuous” values of weights for initial parameters.
• name_model - name of the model: “STRONG”, “WEAK”, “CF_STRONG” or “CF_WEAK”.
• name_init - name of initial clones: “Mutated_cell”, “Thousand_cells” or “Mutated_cell_in_Thousand_cells”.
• ID_Simulation - the identification number of a simulation.
• APC_max_1, APC_max_2, etc. - variant allele frequencies for APC gene with descending order.
• KRAS_max_1, KRAS_max_2, etc. - variant allele frequencies for KRAS gene with descending order.
• TP53_max_1, TP53_max_2, etc. - variant allele frequencies for TP53 gene with descending order.
• PIK3CA_max_1, PIK3CA_max_2, etc. - variant allele frequencies for PIK3CA gene with descending order.

**Table 3:** The results of simulations in the file “data_base_MODELS_PRIMARY.txt”.

name_weights	name_model	name_init	ID_Simulation	APC_max_1	KRAS_max_1	TP53_max_1	PIK3CA_max_1
Discrete	STRONG	Mutated_cell	159	0	0.0	0.5	0
Discrete	STRONG	Mutated_cell	175	0	0.0	0.5	0
Discrete	STRONG	Mutated_cell	222	0	0.5	0.0	0
Discrete	STRONG	Mutated_cell	267	0	0.0	0.5	0
Discrete	STRONG	Mutated_cell	430	0	0.5	0.0	0
Discrete	STRONG	Mutated_cell	522	0	0.5	0.0	0
Discrete	STRONG	Mutated_cell	622	0	0.5	0.0	0
Discrete	STRONG	Mutated_cell	654	0	0.5	0.0	0
Discrete	STRONG	Mutated_cell	663	0	0.0	0.5	0
Discrete	STRONG	Mutated_cell	670	0	0.5	0.0	0

Table 4 shows the first several rows of the dataset for initial parameters for each simulation. It has information about probabilities of the environment death, parameters of sigmoid function for apoptosis death, etc. and also weights between cancer hallmarks and driver genes [2,3]. The file “Initial_parameters_Discrete_ALL.txt” has the same structure but the data are discrete. Table 5 shows how the weights from input file correspond to the hallmarks of cancer. The weights here are a quantitative representation of qualitative dependencies from the dataset of somatic cancer genetics at high-resolution (COSMIC) [3]. For each simulation, the values of weights from Table 5 are written as a row vector in Table 4. The files “Compaction_Factor_Discrete_ALL.txt” and “Compaction_Factor_Continuous_ALL.txt” have the same structure and contain the input data of compaction factors (Table 6). The hallmarks are denoted as an abbreviation, for example, Ha – apoptosis hallmark (see Table 5).

• ID_Simulation - identification number of a simulation.
• Mutated_Gene – the name of a driver gene for an initial cell.
• coefficients and initial probabilities in the simulator tugHall [2]:
  ○ E0 – the environmental variable gives the maximum number for logistic growth as 1/E0,
  ○ F0 – this parameter serves to extend the maximum cell number defined by E0, through angiogenesis,
  ○ m0 – parameter to define the probability of point mutation,
  ○ uo, us – the probabilities that oncogenes and suppressor genes are impaired by point mutations, respectively,
  ○ s0 – coefficient in sigmoid function,
  ○ k0 - the probability of environmental death,
  ○ d0 – the initial probability of division.
• w_Ha_KRAS, w_Ha_TP53, w_Ha_PIK3CA, w_Hb_KRAS, etc. – the weights between cancer hallmarks and genes (kindly see Table 5).

**Table 4:** The dataset of initial parameters from the file “Initial_parameters_Continuous_ALL.txt”.

ID_Simulation	Mutated_Gene	E0	F0	m0	uo	us	s0	k0	d0	w_Ha_APC	w_Ha_KRAS
1	APC	1e-04	10	0	0.5	0.5	10	0.2857143	0.5	0.2331	0.0452
2	KRAS	1e-04	10	0	0.5	0.5	10	0.2857143	0.5	0.2331	0.0452
3	TP53	1e-04	10	0	0.5	0.5	10	0.2857143	0.5	0.2331	0.0452
4	PIK3CA	1e-04	10	0	0.5	0.5	10	0.2857143	0.5	0.2331	0.0452
5	APC	1e-04	10	0	0.5	0.5	10	0.2857143	0.5	0.5064	0.0909
6	KRAS	1e-04	10	0	0.5	0.5	10	0.2857143	0.5	0.5064	0.0909
7	TP53	1e-04	10	0	0.5	0.5	10	0.2857143	0.5	0.5064	0.0909
8	PIK3CA	1e-04	10	0	0.5	0.5	10	0.2857143	0.5	0.5064	0.0909
9	APC	1e-04	10	0	0.5	0.5	10	0.2857143	0.5	0.4810	0.0673
10	KRAS	1e-04	10	0	0.5	0.5	10	0.2857143	0.5	0.4810	0.0673

Columns from 13 to 20 of dataset											
w_Ha_TP53	w_Ha_PIK3CA	w_Hb_KRAS	w_Hb_TP53	w_Hb_PIK3CA	w_Hd_APC	w_Hd_KRAS	w_Hd_TP53				
0.2948	0.4269	0.7011	0.0443	0.2546	0.4575	0.0053	0.4664				
0.2948	0.4269	0.7011	0.0443	0.2546	0.4575	0.0053	0.4664				
0.2948	0.4269	0.7011	0.0443	0.2546	0.4575	0.0053	0.4664				
0.2948	0.4269	0.7011	0.0443	0.2546	0.4575	0.0053	0.4664				
0.0501	0.3526	0.5761	0.3964	0.0275	0.0628	0.4848	0.2487				
0.0501	0.3526	0.5761	0.3964	0.0275	0.0628	0.4848	0.2487				
0.0501	0.3526	0.5761	0.3964	0.0275	0.0628	0.4848	0.2487				
0.0501	0.3526	0.5761	0.3964	0.0275	0.0628	0.4848	0.2487				
0.2829	0.1688	0.0586	0.7028	0.2386	0.4786	0.0091	0.2528				
0.2829	0.1688	0.0586	0.7028	0.2386	0.4786	0.0091	0.2528				

Columns from 21 to last one of dataset											
w_Hd_PIK3CA	w_Hi_KRAS	w_Hi_TP53	w_Him_APC	w_Him_KRAS	w_Him_TP53	w_Him_PIK3CA					
0.0708	0.0448	0.9552	0.1350	0.3402	0.3727	0.1521					
0.0708	0.0448	0.9552	0.1350	0.3402	0.3727	0.1521					
0.0708	0.0448	0.9552	0.1350	0.3402	0.3727	0.1521					
0.0708	0.0448	0.9552	0.1350	0.3402	0.3727	0.1521					
0.2037	0.2881	0.7119	0.5486	0.2305	0.1311	0.0898					
0.2037	0.2881	0.7119	0.5486	0.2305	0.1311	0.0898					
0.2037	0.2881	0.7119	0.5486	0.2305	0.1311	0.0898					
0.2037	0.2881	0.7119	0.5486	0.2305	0.1311	0.0898					
0.2595	0.6228	0.3772	0.1176	0.0958	0.2540	0.5326					
0.2595	0.6228	0.3772	0.1176	0.0958	0.2540	0.5326

**Table 5:** Keys for the weights between hallmarks and genes.

**Table 6:** The dataset of the values of compaction factors from the file “Compaction_Factor_Continuous_ALL.txt”.

ID_Simulation	Ha	Hb	Hd	Hi	Him
1	0.4319	0.4327	0.9394	0.9400	0.5426
2	0.6446	0.7303	0.9352	0.9964	0.2173
3	0.3657	0.3544	0.4082	0.3846	0.9489
4	0.5659	0.2147	0.3671	0.7229	0.6605
5	0.8507	0.8126	0.4048	0.8700	0.1073
6	0.3378	0.1917	0.3279	0.7194	0.1554
7	0.5588	0.8295	0.3076	0.4590	0.5643
8	0.1922	0.6409	0.8168	0.2721	0.3254
9	0.3163	0.7428	0.9025	0.8220	0.5055
10	0.5653	0.1143	0.2902	0.5740	0.6546

## 2 Experimental Design, Materials and Methods

**Fig. 1:**
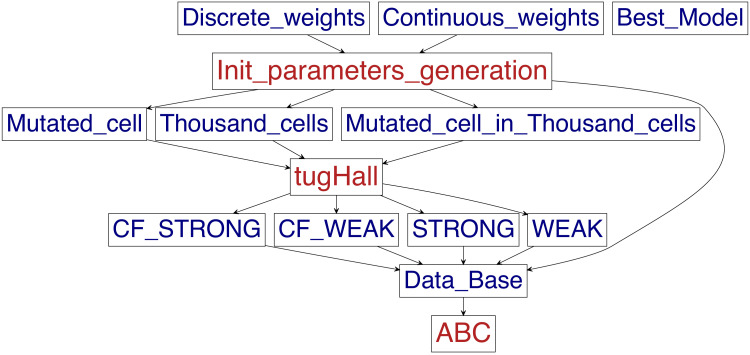
Flowchart of the procedure for simulations, using 4 models, 3 initial conditions and 2 types of values.

Fig. 1 shows the flowchart of the procedure for simulations, using 4 models, 3 initial conditions and 2 types of input data. Therefore, there are 24 types of simulations (Table 2). The procedure includes 4 models with a common part that are fully described in the supplementary materials of the manuscript [2]. There are differences in a few conditions for hallmarks and the initial clones. Models are divided by two criteria:

- The compaction factor *c* - with or without compaction factor. The *c* ∈ [0.1; 1] is compaction factor in the dependencies $H_{x}=c_{x}\cdot \sum_{k=1}^{N_{genes}}g_{k}\cdot w_{k}$, where $g_{k}=1$ when function of gene *k* is destroyed, and $g_{k}=0$ for normal state, $w_{k}$ is a weight for related gene. The index x relates to the hallmarks [4]: $H_{x}=\left\{H_{a},H_{b},H_{i},H_{d},H_{im}\right\}$ and $c_{x}=\left\{c_{a},c_{b},c_{i},c_{d},c_{im}\right\}$.
- The invasion/metastasis transformation condition: $im^{\prime }=1$ (strong condition) or $im^{\prime }>0$ (weak condition).

So, there are 4 models: with and without compaction factors, and with the strong or weak condition of invasion/metastasis transformation (Table 7). Discrete and continuous weights define the value type of weights (Fig. 1). Firstly, the generation of initial parameters occurs with the saving them to the files “Initial_parameters_Discrete.txt” and “Initial_parameters_Continuous.txt”, also the files “Compaction_Factor_Continuous.txt” and “Compaction_Factor_Discrete.txt” include the values of compaction factors for each simulation.

**Table 7:** Relations between models’ names and parameters of models: the condition of invasion/metastasis transformation and presence or absence of compaction factors.

	With compaction factor	Without compaction factor
Condition $im^{{}^{\prime }}=1$	CF_STRONG	STRONG
Condition $im^{{}^{\prime }}>0$	CF_WEAK	WEAK

The dataset has three cases for initial clones:

**CASE I: “Mutated_cell”** with few exceptions, the tumor cell population(s) in a human, including metastatic one, are originated from only one cell (clonal mutations) [5]. So, the clones usually have a single common ancestor. That is why we set one possibility to start from just one primary cell. If we start from 1 cell in simulation, however, the cell population becomes extinct in most cases. In the case of extinction, we have to automatically “restart” the simulation (by default we set 100 as the number of restarting). The restarting function is implemented as an additional part of this case. To accelerate the simulation the initial primary cell should have a driver mutation at one gene (the column “Mutated_Gene” in Table 4).

**CASE II: “Thousand_cells”** another case is to start from 1000 primary cells in order to increase the probability of mutation in one simulation and decrease computational cost. However in this case there are possibly several tumors originated from different normal cells and the tumors do not share any mutations.

**CASE III: “Mutated_cell_in_Thousand_cells”** and the third case is a combination of two previous cases. We start with 1000 primary cells, where one cell has a driver mutation.

The flowchart in Fig. 1 shows the procedure of each simulation. tugHall gets initial parameters and (if it is needed) compaction factors related to simulation ID. The weights for hallmarks-genes relations are generated in accordance with statistics data of the Catalogue of Somatic Mutations in Cancer [3]. Then it chooses the case of the initial cell and a model. After the simulation tugHall saves VAF to the file. Finally, Approximate Bayesian computation (ABC) uses the dataset of VAF to get personalized weights related to the VAF of a patient, for example, in our works [2,6] we used VAF of patients from the open datasets of the Cancer Genome Atlas [7].

For calculations, the supercomputer SHIROKANE was used [8]. To get a large number of simulations, we designed a new version of tugHall v.2.1 [6] that allows to accelerate the calculations up to 10^4^ times in comparison with version 1.0. In R script the parallel library was used, which allows making parallel simulation in one node/computer with many cores. For usage of multiple nodes, an array job was used (one job for each node with parallel simulations at each node). The number of jobs was 40 with 10,000 simulations for each type of simulation. The computational cost was around 44−47 h per job or node with 24 cores. In total 960 processors for 47 h were used for 9.6 million trials of 24 types of simulations.

## Declaration of Competing Interest

The authors declare that they have no known competing financial interests or personal relationships which have, or could be perceived to have, influenced the work reported in this article.
